# The Propagation of Cholera

**Published:** 1855-12

**Authors:** 


### THE PROPAGATION OF CHOLERA.

The theory first advanced by Dr. Snow regarding the mode of propagation of cholera is, with certain modifications, the value of which has been already discussed in this Journal, becoming very widely known and accepted. Dr. Alison of Edinburgh has lately expressed views in the *Edinburgh Medical Journal* regarding the poisonous character of cholera excreta, which are of great interest. On the continent Pettenkofer has published a work entitled "*Mode de Propagation du Cholera*," containing a *resumé* of observations made in Bavaria in 1854. The most important opinion in his work is, *that persons attacked with diarrhœa, even slightly, during a time of epidemic cholera, can propagate the disease.* He illustrates this by the facts, that the 225 attendants of the Palace of Industry (at Munich) propagated the cholera in their respective districts, though they only had slight relaxation; that at Ebrach, the cholera outburst followed the arrival of a prisoner from Munich, who had only slight diarrhœa—the washerwoman who washed his soiled linen quickly falling a victim to cholera; that at Ratisbon the book-keeper of the porcelain manufactory returned from Munich with diarrhœa: ten days afterwards, cholera broke out in his house—he and five lodgers died; that in the prison of Kaiserheim a similar circumstance occurred. The period of incubation is from eight to twenty-one days, according to the greater or less amount of moisture in the atmosphere. The epidemic has a time of increase, a stationary period, a period of decrease, and often a recrudescence, especially if there be an arrival of strangers. The duration of the disease in each house is, on an average, twelve days, maximum twenty-one, minimum two. In districts, the average duration is forty-five days. The author believes that districts built on rock are exempt from cholera; that the quantity of septic matter produced depends on the nature of the soil; and that the composition of the soil, and especially the greater or less humidity of its bed, greatly influence the development of cholera. This development is favoured by commerce. Man leaves to the soil urine and excrements, tainted by the influence of a medium where cholera is prevalent, and possessing from that the property of reproducing a poison—in fact, cholera, provided a favourable soil is met with. The tainted matters undergo, in moist ground, a slow fermentation, producing, besides the usual gases, the choleraic virus. This shows itself in three forms, according to individual organisation—diarrhœa, cholerine, and cholera: the attack occurs especially in the night; non-renewal of air, and the general weakness of the organism during sleep, perhaps also favouring its production in that time. Recrudescence is explained by the arrival in a district of persons who have not, as it were, undergone the test of the cholera poison.

In the production of cholera at sea, the author says that the timber, linen, etc. act the part of the soil. He attributes but little influence to sudden changes of temperature, but considers the humidity to play a very decided part, and the influence of ozone to be very marked as anti-miasmatic.

The substances hindering the fermentation of human excreta are the soluble metallic salts, especially those of iron and zinc. Liebig has proposed sulphatic salts. The author prefers a salt of basic lime. These remedies, however, are prophylactic only, and useless when the germ of the disease is present.

